# A knowledge, attitudes, and practice survey among obstetrician-gynaecologists on intimate partner violence in Flanders, Belgium

**DOI:** 10.1186/1471-2458-6-238

**Published:** 2006-09-26

**Authors:** Kristien Roelens, Hans Verstraelen, Kathia Van Egmond, Marleen Temmerman

**Affiliations:** 1 Department of Obstetrics & Gynaecology, Faculty of Medicine & Health Sciences, Ghent University, Ghent University Hospital, De Pintelaan 185, B-9000 Ghent, Belgium; 2 International Center for Reproductive Health, Faculty of Medicine & Health Sciences, Ghent University, Ghent University Hospital, De Pintelaan 185, B-9000 Ghent, Belgium

## Abstract

**Background:**

Intimate partner violence (IPV) has consistently been found to afflict one in twenty pregnant women and is therefore considered a leading cause of physical injury, mental illness and adverse pregnancy outcome. A general antenatal screening policy has been advocated, though compliance with such guidelines tends to be low. We therefore attempted to identify potential barriers to IPV screening in a context where no guidelines have been instigated yet.

**Methods:**

Questionnaire-based Knowledge, Attitude, and Practice survey among obstetrician-gynaecologists in Flanders, Belgium (n = 478).

**Results:**

The response rate was 52.1% (249/478). Gynaecologists prove rather unfamiliar with IPV and therefore largely underestimate the extent of the problem. Merely 6.8% (17/249) of the respondents ever received or pursued any kind of education on IPV. Accordingly they do feel insufficiently skilled to deal with IPV, yet sufficiently capable of recognizing IPV among their patients. Survey participants largely refute the incentive of universal screening in favour of opportunistic screening and do not consider pregnancy as a window of opportunity for routine screening. They do consider screening for IPV as an issue of medical liability and therefore do not suffer from a lack of motivation to screen. In addition, obstetrician-gynaecologists do believe that screening for IPV may be an effective means to counteract abusive behaviours. Yet, their outcome expectancy is weighed down by their perceived lack of self-efficacy in dealing with IPV, by lack of familiarity with referral procedures and by their perceived lack of available referral services. Major external or patient-related barriers to IPV screening included a perceived lack of time and fear of offending or insulting patients. Overall, merely 8.4 % (21/245) of gynaecologists in this survey performed some kind of IPV questioning on a regular basis. Finally, physician education was found to be the strongest predictor of a positive attitude towards screening and of current screening practices.

**Conclusion:**

Endorsement of physician training on IPV is an important first step towards successful implementation of screening guidelines for IPV. Additional introduction of enabling and reinforcement strategies such as screening tools, patient leaflets, formal referral pathways, and physician feedback may further enhance compliance with screening recommendations and guidelines.

## Background

Intimate partner violence is defined as physical, psychological, economic or sexual coercion of one partner in a relationship by the other [1]. The pattern of abusive behaviours within a relationship afflicts primarily women and, as a ubiquitous phenomenon, thereby crosses the boundaries of culture, religion, and social class [2].

As a leading cause of physical injury, mental illness and adverse pregnancy outcome [3,4] domestic violence is not so much an emerging women's health issue, but rather a continuing hidden epidemic. According to a recent comprehensive review, lifetime-incidence estimates of partner-inflicted harm to women range from 10 up to 69% [3]. In an earlier review on partner abuse during pregnancy, violence in pregnancy was found to occur with 0.9 to 20.1% of pregnancies [5], the preponderance of studies rather consistently indicating a 3 to 8% abuse rate during pregnancy. In Flanders, Belgium we recently obtained an estimated prevalence of physical and or sexual abuse among pregnant women of 3.1% during pregnancy and of 4.4% in the year preceding pregnancy [6].

Overall little progress has been made in tackling what might well be called, women's most imminent health threat. As a straightforward corollary, systematic screening for intimate partner abuse by health care providers including gynaecologists – as the primary guardians of women's wellbeing – has been advocated by several authoritative US bodies, including the American Medical Association [7], the American College of Obstetricians and Gynecologists [2,8], the American Academy of Pediatrics [9], the American College of Emergency Physicians [10], the American Academy of Family Physicians [11] and the Centers for Disease Control and Prevention [8]. Yet, despite these widely promulgated guidelines, it has been acknowledged that screening for domestic violence is not a routine part of most medical visits [1].

In fact, clinical guidelines or recommendations as such generally tend to have little effect on physician's behaviours or practice [12]. This common lack of adherence to clinical practice guidelines has been comprehensively modelled by Cabana *et al *[13], who basically identified a defined number of barriers underlying physician's lack to comply with guidelines.

We modified the construct developed by Cabana et al, which was derived from an extensive literature review [13], as a predictive model to assess current barriers to screening for intimate partner violence in a setting were no clinical guidelines nor specific recommendations with regard to abuse have been instigated. We hypothesized that if all previously identified barriers to guideline adherence [13] could be assessed, guidelines can be more successfully launched in the nearby future by simultaneously accounting for their potential constraints. Barriers with regard to this screening incentive for partner abuse were assessed through a statewide knowledge, attitude, and practice survey among gynaecologists.

## Methods

### Data collection

A questionnaire was basically designed as a knowledge, attitude, and screening and referral practices assessment tool with regard to intimate partner abuse and consisted of 69 items, including 60 items with forced-choice answers (Likert-scale or yes/no) and 9 open-ended questions. The questionnaire has been approved by the Ghent University Hospital Ethical Board and by the Flemish College of Obstetricians and Gynaecologists and was sent to all members of the Flemish College of Obstetricians and Gynaecologists (n = 478). In this statewide survey among board-certified obstetrician-gynaecologists in Flanders (n = 478), a total of 249 completed questionnaires were returned and available for further analysis, corresponding to a response rate of 52.1%. Basic characteristics of survey participants are displayed in table 1.

The questionnaire was divided into seven sections; i.e. **a) **physician and practice characteristics (n = 10), **b) **prevalence of intimate partner abuse (n = 2), **c) **current partner abuse screening practices (n = 7), **d) **attitude towards partner abuse screening (n = 15), **e) **recent assessment, treatment and/or referral of patients in case of intimate partner abuse (n = 19), **f) **knowledge and attitude towards referral possibilities and facilities in case of intimate partner abuse (n = 14), and **g) **willingness and motivation to screen and to participate in future research with regard to intimate partner abuse (n = 2).

**Figure 1:**
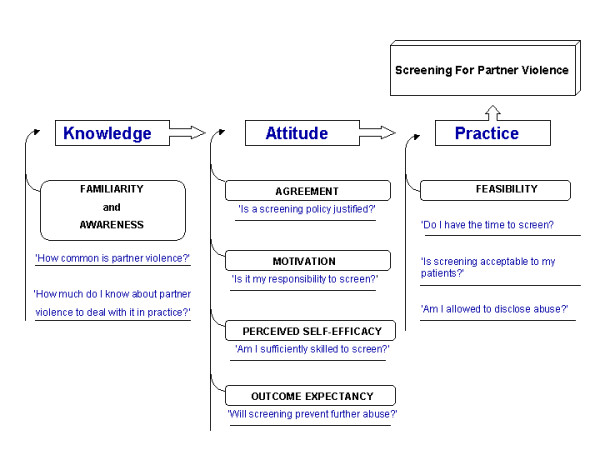
Conceptual framework of knowledge, attitude, and practice as determinants of screening for intimate partner violence.

The questions and survey statements were principally developed to fit a conceptual framework (see figure 1). This behavioural construct was developed by Woolf [14] and subsequently modified by Cabana *et al*. [13] in a systematic review of barriers to physician adherence to clinical practice guidelines. We modified the model, which was derived from a retrospective literature review [13] and applied it as a conceptual framework to model expected barriers to future guideline implementation with regard to screening for intimate partner abuse.

Barriers identified fit into one of three major groups depending on whether they affect physician's knowledge, behaviour, or practice. The model assumes a behavioural framework, i.e. before specific physician-targeted information on a health-related issue modifies patient outcome, it first affects physician's knowledge, then physician's attitude, and eventually physician's behaviour and practice [13]. Though other pathways may be involved, this algorithm is believed to underlie the most sustainable behavioural modification.

Of the surveys reviewed by Cabana *et al *most dealt with one, two, or three barriers [13]; in the present survey we simultaneously accounted for all six major types of barriers retained in the modified model (see figure 1).

### Statistical analysis

Data are primarily presented as the proportion of responders to each question/statement. To assess the impact of a defined set of determinants on the preparedness to screen and on screening behaviour bivariate analyses were performed followed by multivariable analysis using a stepwise binary logistic regression model and likelihood ratio tests were used to compare different models. Performance of the model was reported trough the model chi-square and goodness-of-fit was assessed by the Hosmer and Lemeshow test (goodness-of-fit chi-square). The beta-values obtained from the multivariate model are presented as adjusted odds ratios (AOR) with 95% confidence intervals (CI) and p-values to the 95% CI. Statistical significance was accepted, as the two-tailed probability level was <0.05.

Data-entry was performed with the statistical software package Epi Info v. 6.04 and all statistical analyses were performed with he statistical software package SPSS v. 12.0 (Chicago, Illinois).

## Results

### Knowledge: familiarity and awareness

#### Familiarity with intimate partner abuse

Merely 6.8% of the respondents in the survey (17/249) stated having received or pursued any kind of education or information on intimate partner violence. Over two thirds (67.9%) of the participating gynaecologists (169/249) acknowledge that there is a defined need to incorporate such education during medical training.

We did not further assess physician's familiarity with risk factors, signs, symptoms, and comorbidity patterns relating to partner violence as an issue of knowledge. Nor did we make an attempt to assess obstetrician's knowledge of screening strategies as an indicator of their relevant knowledge.

#### Awareness of intimate partner abuse

Intimate partner abuse is deemed a rather rare phenomenon by most survey participants, i.e. affecting less than 1 in 100 or even less than 1 in 1000 patients attending. Merely one in four gynaecologists estimated the prevalence of abuse among non-pregnant women to be at least one percent. Similarly, intimate partner abuse is thought to occur with at least one percent of pregnant women by only one in five gynaecologists.

### Attitude: incentive agreement, motivation, perceived self-efficacy, and outcome expectancy

#### Agreement with the incentive to screen

In the main, survey participants decline universal and systematic screening and also refute the common view of pregnancy as a window of opportunity to screen (see table 2). Rather, obstetrician-gynaecologists do favour direct questioning of the patient by means of the Abuse Assessment Screen [15] in case of suspected abuse (see table 2).

#### Motivation

Most physicians surveyed consider directed screening though not universal screening as an issue of medical liability (see table 2). Respondents largely contradict common beliefs about partner abuse and in particular they do not consider it a family affair, for which partners should take responsibility, nor a phenomenon pertaining to lower social classes or an affliction for which the victim itself is to blame (see table 2).

#### Perceived self-efficacy

The preponderance of survey participants feels insufficiently skilled to discuss partner abuse in a straightforward manner and to manage abuse-related issues with putative victims of domestic violence (see table 2). Similarly, physicians surveyed feel insufficiently acquainted with referral practices in case of partner abuse (see table 2).

#### Outcome expectancy

A majority of obstetrician-gynaecologists believes that screening for intimate partner violence may be an effective means to counteract such abusive behaviours (see table 2). Yet, about half of all survey respondents are also convinced that there is a defined lack of referral services and specialised care facilities for women suffering from domestic violence (see table 2).

### Practice and behaviour: screening practices and perceived barriers

When asked for current screening practices at the time of the survey very few obstetricians appear to adhere to a universal screening policy. Merely 8.4 % of all participants (21/249) stated to screen each patient at least once during pregnancy (see table 3). Lack of time and fear of offending or insulting patients were the most frequently cited barriers towards implementation of screening (see table 4).

Survey respondents feel confident in relying on their clinical index of suspicion in their screening practice (results not shown) and stated to launch direct questions most of the time in case of suspected abuse (see table 4). Partner abuse is however only suspected in case of overt physical laesions, whereas psychological distress or complaints more rarely prompt direct questioning about potential abusive behaviours (see table 4).

An indirect estimate of screening sensitivity was obtained from a series of survey questions regarding the most recent case of physical and/or sexual abuse treated by the responding physicians (*results not shown*). The median time span elapsed at the time of the survey since the last victim encounter was 6.3 months (interquartile range 3.1 to 12.8 months) and 5.9 months (interquartile range 2.3 to 12.4 months) for sexual abuse and physical abuse respectively. Overall, one in three obstetricians stated not to have encountered sexual coercion and two in three not to have confronted physical abuse among their patients over the past five years.

### Determinants of attitudes, practice and behaviour

To identify potential determinants of physician's attitude and behaviour with regard to screening for intimate partner violence we applied binary logistic regression analysis. We assumed that the statement "Are you prepared to screen with the Abuse Assessment Screen form each and every patient" is the main outcome variable of the obstetrician's attitude towards screening whereas the question "Do you screen every patient at least once during pregnancy?" is the main outcome variable reflecting obstetrician's screening behaviour. In these models the following physician-related independent variables were accounted for: age (< 40 versus ≥ 40 years), gender, time elapsed since board certification as an obstetrician-gynaecologist (<10 versus ≥ 10 years), practice volume indices (< 250 versus ≥ 250 patients a month and <10 versus ≥ 10 deliveries a month), workload (< 40 versus ≥ 40 hours a week), education/training on intimate partner violence (yes/no), primary hospital type (public, private or university hospital), and having a peer with a known history of abuse (yes/no). Cut-off values for age, years elapsed since board certification, physician's practice volume, and workload were chosen as the 25^th ^percentile of each distribution of these values.

#### Attitude: preparedness to screen

Agreement with the AAS screening incentive was significantly positively associated with lower volume of patients attending (p = 0.008), lower number of deliveries to perform (p = 0.045), having a peer that is a victim of partner abuse (p = 0.012), and a history of having received or pursued any kind of education or information on intimate partner violence (p = 0.019), and marginally significant associated with hospital type (p = 0.052). In the multivariable model lower volume of patients attending (AOR = 2.76, 95% CI: 1.21 – 6.33), a history of specific training or education (AOR = 4.42, 95% CI: 1.18 – 16.67) and having a peer that is a victim of partner abuse (AOR = 2.81, 95% CI: 1.15 – 6.85), were all significant predictors of the preparedness to adhere to a universal screening policy. Overall the final multivariable model performed well with a fairly goodness-of-fit (model χ^2 ^= 15.8, p = 0.001 and goodness-of-fit χ^2 ^= 1.62, p= 0.445).

#### Practice: current screening practices

Of all potentially related variables only a history of specific training or education was significantly associated with the practice of screening each patient during pregnancy (p < 0.001). In the multivariable analysis physician training or education was also significantly and very strongly associated with adherence to a universal screening practice (AOR = 12.48, 95% CI: 3.68 – 42.30), but the model failed to adequately fit the data (model χ^2 ^= 14.09, p < 0.001 and goodness-of-fit χ^2 ^= 0). Therefore, it can only be stated that of all determinants explored, only physician education of or training was associated with screening every patient at least once during pregnancy in a bivariate analysis (crude OR = 10.80, 95% CI: 3.57 – 32.67), whereas none of the other potential determinants did.

## Discussion

### Agreement with a routine screening policy

Despite growing concern about intimate partner violence as a major public health issue, obstetrician-gynaecologists in our survey largely underestimate the extent of the problem of intimate partner violence, disprove for the most part a universal screening policy, and accordingly, very few of them tend to screen their patients on a regular basis. It may therefore be reiterated that many medical organizations and domestic violence experts do recommend routine screening as the first step in the intervention process for domestic violence because of the prevalence of domestic violence, because routine screening allows for early identification of domestic violence, because knowing about the abuse has potential value in the care of the patient, and because of the low risk of harm in screening [16]. While most clinicians in our survey only ask their patients for potential exposure to abuse in case of overt laesions and physical injuries, the challenge for health care professionals is therefore to move toward secondary prevention, i.e. routine screening for intimate partner violence whether or not symptoms are immediately apparent. From a public health perspective, physicians involvement in universal screening for domestic violence may fit a broader framework of preventive strategies that encompasses primary as well as secondary and tertiary prevention [17-19]. In Flanders, Belgium obstetrician-gynaecologists not only account for gynaecologic and obstetric pathology but also act as the primary care physicians to the general female population, e.g. in providing primary obstetric care and in offering preventive women's health medicine. Hence, this setting provides obstetrician-gynaecologists with a unique and broad-coverage detection opportunity, which should allow them to be proactively involved in the secondary prevention of intimate partner abuse. At present, no recommendations regarding screening for domestic violence have been made however in this health care setting nor by governmental and public health authorities, neither by professional medical organisations such as the Flemish College of Obstetricians and Gynaecologists.

### Barriers to routine screening

In the present study we aimed to assess a wide array of barriers to routine screening for intimate partner violence through a statewide knowledge, attitude, and practice survey among obstetrician-gynaecologists. It was hypothesized that barriers would fit an explanatory model which assumes that physician-targeted information on intimate partner violence first modifies physician's knowledge, then physician's attitude, and eventually physician's behaviours and practice [13]. Though less than ten percent of the survey participants received or pursued any kind of education or information on intimate partner violence, we established that physician education was indeed the strongest predictor of a positive attitude or willingness to screen each and every patient and of current screening practices for domestic violence.

Major intrinsic barriers to routine screening included a lack of awareness, as obvious from a striking underestimation of the extent of the problem by most participants, and a defined perceived lack of self-efficacy, in particular the inability to skilfully discuss, manage, and refer (potential) cases of domestic violence. Extrinsic barriers on the other hand, including a perceived lack of time and perceived inappropriateness of questioning patients about partner abuse further compromised obstetrician's screening attitude and practice.

These findings are largely in accordance with previous studies indicating that physicians found exploring domestic violence in the clinical setting analogous to "opening Pandora's box" [20]. In a comprehensive review of published studies on screening for intimate partner violence by health professionals [21] lack of provider education and lack of time were also among the most commonly cited barriers. Important patient-related external barriers in previous studies were also anticipated patient nondisclosure and anticipated patient fear of repercussions [21,22], two issues that were not addressed in our study. Patient nondisclosure much alike the anticipated fear of offending or insulting patients observed in our study may result from a longstanding misconception about patient's beliefs and expectations, as we recently assessed that the vast majority of women from a general obstetric population actually do favour direct questioning about intimate partner violence by their gynaecologists, regardless of a history of partner abuse [6].

Anticipated patient fear of repercussions is a more intricate issue, as it has not been firmly established from an evidence-base principle that a screening policy does not endanger women and their children at risk of experiencing abusive behaviours. As a matter of fact it has been suggested that screening for violence could be harmful, for example by causing psychological distress or by leading to a further escalation of abuse [23]. While many others have contradicted this view [16] it must be acknowledged that, at the very least, future studies are still warranted to assess how patient safety can be ensured when a universal screening policy is to be applied.

Finally our study is less in agreement with the preponderance of previous studies in which lack of effective interventions or poor outcome expectancy was found as a commonly cited barrier [21,24]. Gerbert *et al *for instance documented in a survey among primary care physicians that interventions for domestic violence were deemed less successful as compared to interventions for other major public health challenges such as tobacco control and HIV/STD risk behaviour interventions [16]. In our survey, obstetricians generally do not share the fatalistic view that there is no solution for the problem anyway, even though they tend to report a perceived lack of referral services and specialised care facilities for women suffering from domestic violence. Along with their positive outcome expectancy, which is definitely a cornerstone to the implementation of a successful screening policy, it further appears as if medical liability and ethical duty further adds to the screening motivation of respondents in our survey. Though it could be argued that medical liability and ethical duty leaves physicians little room for opting out in this matter, this issue seems to be meagrely addressed in the literature. It is further of note that, in contrast to several previous surveys, obstetricians in our study refuted common misbelieves about partner abuse, in particular they did not consider partner violence as a family affair, for which partners should take responsibility, nor as a phenomenon pertaining to lower social classes or an affliction for which the victim itself is to blame.

### Helping physicians in adopting a screening strategy: predisposing, enabling, and reinforcing strategies

As was hypothesized at the outset, we found that physician education was significantly associated with screening attitude and behaviour and interestingly, that having a peer with a history of abuse, which might be viewed as a proxy for familiarity with the issue, also affects physicians' proneness to screen. These findings seem to support the paradigm that increasing knowledge will enhance a positive attitude towards screening and therefore actual screening practices [13], though this view has been challenged in at least one study [25].

If anything, it may be acknowledged from intervention studies that instigating 'predisposing' strategies (e.g. education) as such tend to have little effect on eventual physician's behaviours [21] and hence on their compliance with clinical guidelines. Additionally providing physicians with 'enabling' tools (e.g. screening tools such as the AAS) however, has been found more effective in changing health care provider's behaviours. The use of additional 'reinforcement' strategies such as providing physicians with feedback with regard to their screening practices may further amplify the process of behavioural change [26-28].

Accordingly, it may be inferred that such 'predisposing', 'enabling', and 'reinforcing' strategies may supersede most barriers identified, e.g. the external barrier of time and the internal barrier of perceived self-efficacy, which might no longer be a constraint when provided with an easy-to-handle, time-efficient and acceptable routine screening tool, such as the AAS. 'Enabling' and 'reinforcement' strategies are also likely to help physicians in lowering their threshold for asking questions about partner abuse, to enhance their motivation and to increase their satisfaction with clinical practice.

The crux of intimate partner violence is really that most women who encounter some kind of coercion will not present with overt signs of abuse, but rather with a wide variety of vague and non-specific symptoms, if any. The physician's eye is therefore even in the presence of a high index of clinical suspicion unlikely to grasp most victims and their potential signs in a general obstetric or gynaecologic population. This was also apparent from the present survey. First of all, obstetrician-gynaecologists revealed that clinical detection of violence primarily depends on the presence of physical trauma, whereas psychological or psychosomatic complaints rarely are the impetus to direct questioning about abuse. Secondly, one in three obstetricians stated not to have encountered sexual coercion and two in three not to have confronted physical abuse among their patients over the past five years. Since we previously found an estimated prevalence of physical and or sexual abuse among pregnant women of 3.1% during pregnancy and of 4.4% in the year preceding pregnancy [6], obstetricians in this survey were actually expected to see at least one patient experiencing partner abuse a month.

Of note is that the optimal mode of administration of screening to detect partner abuse remains uncertain. A number of screening tools have been proposed [15,29], but few comparative analyses on these are available. Since gynaecologists in our survey strongly opposed to direct screening with the AAS form, an alternative that might be considered is to provide the woman with the opportunity to self-disclose partner violence using a checklist [30], such as the Maternal Social Support Scale [31]. As part of the registration process with the latter, the pregnant women have to complete a Maternity Social Support Scale including two items relating to partner violence: "I feel controlled by my husband/partner" and "There is conflict with my husband/partner" [31].

### Does routine screening result in better patient outcomes?

Whereas poor outcome expectancy was found as a commonly cited barrier in previous studies [24], it is definitely reassuring that the preponderance of obstetrician-gynaecologists in our survey do believe that screening for intimate partner violence may be an effective means to counteract such abusive behaviours. Glowa *et al *previously documented that physicians who identified victims of intimate partner violence after screening indeed reported more often positive patient outcomes (e.g. improved mental health, seeking counselling or services) than negative outcomes (e.g. worsening of violence, substance abuse) and that the physicians believed that these outcomes primarily resulted from disclosure [32]. Similarly, the adagio that "Recognising the problem of partner violence as such is definitely the first step towards a possible solution" [16] seems to be a mechanism that has been corroborated by the finding that even brief discussions with a physician, conducted in a concerned and non-judgemental fashion, can help to change the way abused women view their situation, even if they do not disclose the abuse [33]. It may further be stressed that disclosure of violence improves the patient-provider relationship in terms of communication and of patient and provider satisfaction [4,32].

Yet, the effectiveness of a screening policy has been challenged, i.e. it has been put in doubt whether actively identifying partner abuse eventually leads to better patient outcomes. A balanced appraisal of the literature shows that there are actually very few reports on the effectiveness of screening for intimate partner abuse. Though the only systematic review on this subject [34] discouraged a universal screening policy, it may also be acknowledged that the latter review did not include a single randomised controlled trial on screening for intimate partner violence. While such unbiased studies are therefore definitely warranted, several authoritative publications in the JAMA for instance, have advocated that screening for one of the most imminent health treats to women in our society should not await the proof of evidence-based medicine [33,35], as it generally assumed that the benefits of identifying partner abuse are very likely to outweigh its potential drawbacks for both victims and their perpetrators.

Finally, it must be acknowledged that intimate partner violence is strongly associated with other abusive behaviours and child abuse in particular. About half of all children living with mothers suffering from partner abuse will also be exposed to abuse. Intimate partner violence has been associated with severe childhood dysfunction and long-term adverse health effects, e.g. child abuse but also witnessing domestic violence has emerged as a risk for alcoholism, illicit drug use, depressed affect and even suicide [36,37]. Accordingly, the American Academy of Paediatrics has recommended intervening on behalf of battered women as an active form of child abuse prevention [9].

### Study limitations

Major study limitations include the use of forced-choice answers, thereby also potentially obliterating barriers yet to be assessed. Though we addressed all six major types of barriers retained in the modified Cabana model [13] as the conceptual framework of our study, other authors have pinpointed some important potential barriers to screening for domestic violence that we have not accounted for. As discussed above for instance, a limitation to our study is that we did not assess patient nondisclosure, patient safety and patient fear of repercussions. Similarly we failed to account adequately for providers' safety, though we asked if they were anxious to lose control in discussing partner abuse with their patients. Other, yet-to-be-identified determinants undoubtedly may also interfere with physicians' screening attitudes and behaviour. This was also illustrated by the failure of our multivariable analysis in which the nine key determinants included, failed to adequately describe observed screening practices, though there was a strong correlation with physician education. Though the response rate was fairly high in comparison with most similar studies, it should further be acknowledged that the results of our survey should be taken with caution, as almost half of obstetrician-gynaecologists of the sampling frame did not participate in the study. It can therefore not be ruled out that selection bias biased our results, though we established that the sample was representative in terms of gender (sample female/male sex ratio = 65.3 (39.5%,/60.6%) versus 59.0 in the cohort (37.1%/62.9%), Mann-Whitney U test p = 0.522) and age (sample mean = 45.3 years, SD = 10.2 versus cohort mean = 44.2 years, SD = 10.7, independent samples t-test p = 0.18).

Though we designed our study according to the knowledge-attitude-behaviour construct, it must be acknowledged that assessment of knowledge in our survey was limited to some indicators of awareness and familiarity, while the survey did not entail direct questions on risk factors, signs, symptoms, and comorbidity patterns relating to partner violence as an issue of knowledge. Nor did we make an attempt to assess obstetrician's knowledge of screening strategies. Short *et al *also emphasized that many standardised intimate partner violence survey tools do not adequately assess actual knowledge [38]. By accounting for a number of knowledge-related items, Short and colleagues very recently developed a reliable survey instrument – the so-called PREMIS tool (Physician Readiness to Manage Intimate Partner Violence Survey) – that can be used to measure the effectiveness of intimate partner violence educational programs [38].

Several models other than the knowledge-attitude-behaviour model applied in our survey have indeed been developed to assess health care provider characteristics and training needs in relation to intimate partner violence. Of particular interest are those models constructed through the use of psychometric techniques, which have resulted in some refined tools that may guide future IPV policy interventions and training programs [38,39].

It needs to be stressed that in contrast to our study, which was primarily designed as a observational study to identify and quantify a defined set of external and internal barriers to intimate partner violence screening in a health care setting where routine screening is rather an exception to the rule, the aforementioned psychometric models do not only serve as explanatory models, but also allow for monitoring future intervention studies through publicly available instruments derived from these models [38,39].

## Conclusion

Obstetrician-gynaecologists in our survey largely underestimate the extent of the problem of intimate partner violence. Women's physicians also refute for the most part a universal screening policy. Major barriers to a universal screening policy include lack of time, fear of offending patients, and insufficient knowledge of referral facilities.

Obstetrician-gynaecologists do rather favour opportunistic screening for intimate partner abuse. However, as clinical detection was further found to rely primarily on the presence of overt physical trauma, it may be concluded that such opportunistic screening is likely to have a particularly low coverage, leaving most cases of partner violence unrecognised.

Endorsement of physician training on intimate partner violence is an important first step towards successful implementation of screening guidelines for intimate partner violence. Physician education alone is in itself however a necessary, but insufficient answer to all barriers identified. In particular, additional introduction of enabling and reinforcement strategies such as screening tools, patient leaflets, formal referral pathways, and physician feedback may further enhance compliance with clinical guidelines on intimate partner violence.

## Competing interests

The author(s) declare that they have no competing interests.

## Authors' contributions

KR, HV, KV, and MT were involved in designing the study, in the conduct of the study, and participated in the data-collection and analysis. KR and HV drafted the manuscript and KV and MT provided important intellectual content. All authors read and approved the final manuscript.

## Pre-publication history

The pre-publication history for this paper can be accessed here:



## Figures and Tables

**Table 1:** Basic characteristics of survey participants (n = 249)

Age (years) – median (quartile range)	43.5	37.0 to 52.8
		
Sex		
Male – n (%)	60.2	
Female – n (%)	39.8	
		
Numbers of years in clinical practice – median (quartile range)	13.0	6.0 to 21.0
Number of outpatient contacts/month – median (quartile range)	395	250 to 475
Number of deliveries/month – median (quartile range)	15	10 to 20
		
Hospital type		
Public hospital	24.2	
Private hospital	65.9	
University hospital	17.0	

**Table 2:** Attitudes about intimate partner violence among survey participants (n = 249)

Survey statement	Agree (%)	Neutral (%)	Disagree (%)
**INCENTIVE AGREEMENT**			
Are you prepared to screen with the Abuse Assessment Screen form			
Each and every patient (universal screening)?	15.7	14.8	69.5
In case of suspected abuse (directed screening)?	82.7	10.4	6.8
Do you agree that pregnancy provides a unique window of opportunity for (universal) screening?	24.1	27.3	48.6
Do you agree that all pregnant women should be screened at least once during each trimester?	5.2	14.1	80.7
Do you feel that universal screening for partner abuse is not justified because of its low prevalence?	39.3	23.3	37.3
**MOTIVATION**			
Do you agree that gynaecologists have the medical responsibility to screen			
Each and every patient (universal screening)?	15.2	29.7	55.0
In case of suspected abuse (directed screening)?	79.6	9.6	10.8
Do you consider partner violence a family affair for which the partners themselves should take responsibility?	10.4	13.3	76.3
Do you think that partner abuse is a phenomenon pertaining to social disadvantaged groups and therefore screening should be confined to the latter?	7.6	14.5	77.9
Do you agree that women may harass their spouses so badly/to the extent that is conceivable for husbands to lose their temper?	6.4	24.9	68.7
**PERCEIVED SELF-EFFICACY**			
I feel familiar with this issue and I am sufficiently skilled to launch direct questions regarding partner abuse	22.9	25.7	51.4
I am awe to lose control over the situation, once the issue of partner abuse has been addressed	24.1	25.7	50.2
Overall, obstetricians or gynaecologists are insufficiently familiar with partner abuse in order to adequately deal with it and to properly refer patients	60.3	20.5	19.2
As a gynaecologist, I am familiar with the referral possibilities and facilities following the assessment of partner abuse	28.1	12.0	59.8
**OUTCOME EXPECTANCY**			
It is of no use to screen for partner violence, because there is no solution for the problem anyway	12.4	22.1	65.4
It is of no use to screen for partner violence, because of a lack of referral facilities that can provide adequate and specialized care?	44.9	24.9	30.1
Patient referral in case of partner abuse makes no sense, because most abused women will stick to their relationship either which way	26.5	36.1	37.3
If victims of partner abuse charge a complaint with the police, little, if anything, is done about it	37.2	41.8	20.9

**Table 3:** Current practice of intimate partner violence screening among survey participants (n = 249)

	Rarely (%)	Inconsistently (%)	Regularly (%)
Direct questioning about (partner) abuse in case of (directed screening)			
Physical laesions*	11.6	16.5	68.7
Psychological complaints*	51.4	27.7	19.3

**Table 4:** Practice of intimate partner violence screening among survey participants (n = 249): potential limits of feasibility

Survey statement	Agree (%)	Neutral (%)	Disagree (%)
I don't have the time to thoroughly discuss relational problems with victims of partner abuse	57.1	16.5	26.5
Most patients would feel offended or insulted if I would ask them if they have experienced partner abuse	50.6	22.5	26.9
Patient confidentiality and liability prevent me to officially disclose partner abuse	8.8	26.5	64.6
Disclosure of partner abuse through a written statement is tricky and will inevitably become cumbersome	39.3	31.7	28.9
